# The Adolescent Indication for Buprenorphine: What It Is, What It Is Not, and Why It Matters

**DOI:** 10.7759/cureus.118912

**Published:** 2026-10-31

**Authors:** Arya Babul, Parisa Mahdavi

**Affiliations:** 1 Biomedical Sciences, West Career and Technical Academy, Las Vegas, USA; 2 Biomedical Sciences, Society for Awareness of Neglected Diseases, Las Vegas, USA; 3 Drug Regulatory Affairs, Tavolar LLC, Las Vegas, USA

**Keywords:** adolescents, buprenorphine, cascade of care, epistemic injustice, fentanyl era, medication access, opioid use disorder, pediatric labeling, regulatory barriers, treatment disparities

## Abstract

Adolescents with opioid use disorder remain the group least likely to receive medication treatment despite facing the most severe risks in the current fentanyl landscape. Recent discussions in the clinical literature highlight persistent gaps in treatment initiation and engagement among young people, yet many analyses rely on age classifications and regulatory interpretations that do not align with established pediatric drug development standards. In the United States, buprenorphine carries no formal approval for individuals younger than 18 years, even though international authorities recognize adolescent indications and multiple federal bodies support its use in youth. Long‑standing misconceptions about an age‑16 approval, combined with the common practice of merging adolescents and young adults into broad “youth” categories, contribute to structural and epistemic barriers that restrict access to evidence‑based care. Clarifying regulatory terminology and correcting these entrenched misunderstandings are essential steps toward improving buprenorphine access for adolescents in the fentanyl era.

## Editorial

In a recent JAMA Network Open original investigation, Hadland et al. [1] used comprehensive Medicaid data from all 50 states and Washington, DC, to report gaps in the cascade of care for youths with opioid use disorder (OUD). The study examined progression through key treatment stages among individuals aged 13-25 years from 2016 to 2023, reporting on the proportion advancing at each step, identifying disparities across demographic groups, and receipt and retention of medications for OUD (MOUD).

Hadland et al. [1] should be commended for their timely report. Their cascade of care findings are striking: only 50.7% of youth initiated treatment within 14 days of diagnosis, 32.9% engaged in care, and just 15.2% received MOUD [1]. Most concerning, only 6.6% of adolescents received MOUD, compared with 51.2% of young adults. These data underscore the profound risks adolescents face in a fentanyl-dominated drug supply. Their analysis also documents substantial disparities by age and race, with younger adolescents and racially minoritized youths showing consistently lower progression through every stage of the cascade of care.

Similarly, in a narrative review by Ball et al. [2] recently published in JAMA Pediatrics, the authors summarize “current knowledge of opioid use, overdoses, and OUD among US adolescents in the context of fentanyl” and outline the clinical roles of naloxone and buprenorphine for overdose reversal and treatment. Their stated objective is to synthesize contemporary evidence on adolescent overdose and OUD and to describe management strategies “in the era of synthetic opioids,” with particular attention to the practical considerations clinicians face when treating youth. The review also aims to support ongoing efforts to expand access to evidence‑based care by distilling key clinical principles for using naloxone and buprenorphine during a period marked by unprecedented fentanyl‑related risk. Ball et al. further emphasize that adolescents are uniquely vulnerable to fentanyl‑related harms due to developmental factors, limited prior opioid exposure, and frequent underestimation of overdose risk.

Ball et al. [2] should similarly be commended for highlighting the profound gaps in prevention and treatment experienced by adolescents, including the sharp rise in morbidity and mortality driven by fentanyl despite the availability of safe and effective medications for overdose reversal and OUD treatment. Their review also notes recent regulatory changes that expanded access to naloxone and buprenorphine, further highlighting the incongruity between evolving policy and the persistent absence of an adolescent buprenorphine indication.

Hadland et al.’s cohort study is methodologically sound and Ball et al.’s review appropriately comprehensive, though several points in each manuscript warrant clarification.

First, Hadland et al. group individuals aged 13-25 years under broad labels such as “youths,” “adolescents and young adults,” or “adolescents younger than 18 years” [1]. Similarly, Ball et al. refer to "adolescents younger than 18 years" [2]. This practice is inconsistent with regulatory definitions that govern pediatric drug development. As summarized in a recent review by Babul et al., all major regulatory agencies, including the U.S. Food and Drug Administration (FDA), the UK Medicines and Healthcare products Regulatory Agency, the European Medicines Agency, Japan’s Pharmaceuticals and Medical Devices Agency, and China’s National Medical Products Administration, define adolescents as individuals aged 12-17 years [3]. The FDA's Clinical Pharmacology Considerations for Pediatric Studies [4], Pediatric Study Plans guidance [5], the ICH E11(R1) framework [6], and FDA's Pediatric Drug Development: Regulatory Considerations [7] all rely on this demarcation. As noted in that review (Babul et al., 2026), this age range is widely used in FDA-approved prescribing information (PI) to delineate adolescent indications, stratify safety and efficacy data, and structure pediatric labeling [3].

This is not merely a semantic distinction. Collapsing adolescents with young adults may increase sample size, but it obscures age-specific outcomes and perpetuates the evidence gaps that limit adolescent access to MOUD. The absence of an adolescent indication remains a central barrier, shaping clinician hesitancy, influencing family reticence, and steering guideline development and research priorities. It also contributes to payer-driven barriers such as coverage restrictions, reimbursement limitations, and prior authorization delays, while reinforcing longstanding misconceptions and discomfort around treating youth with the same evidence-based medications routinely offered to adults. As Babul et al. describe, these patterns reflect broader epistemic barriers that shape whose evidence is recognized and whose treatment needs are systematically overlooked.

Second, Hadland et al. state that “Buprenorphine is approved by the U.S. FDA for individuals aged 16 years and older” [1], while Ball et al. similarly state “Buprenorphine is the medication most used in youth and the only MOUD that is US FDA-approved for adolescents aged 16 years and older” [2]. This is a widely repeated but incorrect assertion. Among youths who did receive MOUD, buprenorphine was by far the most commonly initiated medication, underscoring the importance of accurate regulatory framing around its adolescent indication. As detailed in the Babul et al. paper, no buprenorphine formulation is FDA-approved for the treatment of OUD in individuals younger than 18 years [3]. Since approval in 2002, the transmucosal buprenorphine PI, also referred to as the label, has stated that these products are “used to treat adults addicted to opioid...” [8]. However, until 2011, the label also noted that “safety and effectiveness … in patients below age 16 have not been established,” which may have been misunderstood by some as approval from age 16 onward [9]. In 2011, the age‑16 wording was removed from the full prescribing information (FPI) [9]. However, this information was inadvertently retained in the highlights of prescribing information (HPI), even though language in the HPI is required to be sourced entirely from the FPI [10]. Nonetheless, this error was eventually corrected with the February 2, 2018, label, when the statement “safety and effectiveness ... in patients below the age of 16 have not been established” was removed in its entirety [11]. The 2018 revision to the pediatric language occurred without publicly available regulatory correspondence or clarification, a lack of transparency that likely contributed to the persistence of the age‑16 misstatement across guidelines and secondary literature.

This misinformation appears in highly influential sources, including the American Academy of Child and Adolescent Psychiatry’s guideline [12], the American Society of Addiction Medicine’s National Practice Guideline [13], American Academy of Pediatrics publications [14], and the Journal of the American Academy of Child and Adolescent Psychiatry’s Revised Principles and Practice Recommendations [15], and has now become entrenched in the secondary literature. The persistence of this error continues to shape clinician behavior long after the underlying regulatory language changed.

International regulatory agencies highlight the magnitude of this misstatement. In the EU and UK, transmucosal buprenorphine is indicated for adolescents aged 15 years and older, and extended-release buprenorphine for adolescents aged 16 years and older. The U.S. is the outlier, not because of evidence, but because of regulatory inertia and persistent miscommunication. By inaccurately stating that buprenorphine is “for individuals aged 16 years and older,” the authors inadvertently legitimize barriers that keep U.S. adolescents from receiving lifesaving medication. Federal agencies, including the National Institute on Drug Abuse and the Substance Abuse and Mental Health Services Administration, explicitly endorse buprenorphine for adolescents with OUD, further underscoring that the persistent treatment gap reflects structural and regulatory barriers rather than any lack of evidence. This regulatory stagnation is precisely why the proposed Estrogen Replacement and Atherosclerosis Trial is long overdue, provided it enrolls a sufficient number of adolescents to permit meaningful age‑stratified analyses [3].

Accurate regulatory framing is essential. Misstatements about adolescent indications shape clinician hesitancy, payer restrictions, and treatment access, and these are precisely the barriers Hadland et al. have long documented. Their important national analysis would be strengthened by aligning terminology with regulatory standards and clarifying the true status of buprenorphine indications for adolescents.

The undertreatment described by Hadland et al. and Ball et al. is not benign; it produces predictable and preventable harms, including overdose, death, and the entrenchment of chronic opioid addiction during adolescence in a fentanyl dominated drug supply [1,2]. Two decades of evidence reveal a clear and persistent pattern: youth with OUD are the population least likely to be offered MOUD, least likely to maintain treatment, and most likely to be routed into nonpharmacologic care, even though they bear the greatest overdose burden and have the most to gain from timely medication initiation. The literature points to a familiar set of interlocking drivers: clinician resistance, hesitation among families and adolescents, structural constraints, regulatory confusion, and developmental challenges that together undermine continuity of care. Adolescents continue to be directed toward psychosocial interventions and short-term detoxification, approaches repeatedly shown to be insufficient. Surveys of addiction treatment staff further illustrate this gradient: enthusiasm for MOUD declines sharply for adolescents aged 16-17 years [16].

A clearer regulatory foundation, coupled with recognition of these epistemic dynamics, would sharpen the insights offered by both Hadland et al. and Ball et al. and help ensure that adolescents receive the evidence based treatment that the current drug supply demands.
